# Seagrass and hydrographic data for the Mediterranean Sea

**DOI:** 10.1016/j.dib.2019.104286

**Published:** 2019-07-19

**Authors:** Dimitrios Effrosynidis, Avi Arampatzis, Georgios Sylaios

**Affiliations:** a Database & Information Retrieval Research Unit, Department of Electrical & Computer Engineering, Democritus University of Thrace, Xanthi 67100, Greece; b Lab of Ecological Engineering & Technology, Department of Environmental Engineering, Democritus University of Thrace, Xanthi 67100, Greece

**Keywords:** Seagrass data, Mediterranean sea, Hydrographic data, Machine learning, Classification, Multi-class

## Abstract

The dataset includes 1,771 locations of major seagrass families (*Cymodoceaceae, Zosteraceae, Posidoniaceae, Hydrocharitaceae, Ruppiaceae*), which are further divided into the species they include, as well as 1,284 locations of seagrass absence (algorithmically produced), in the Mediterranean Sea. For each location, 217 biological, chemical, physics, and human related parameters are available, which were merged from other publicly available data sources. As the most comprehensive dataset for seagrass in the Mediterranean to date, it is suitable for data analysis and machine learning. For more insight, please see “Seagrass Detection in the Mediterranean: A Supervised Learning Approach” (Effrosynidis et al., 2018). The dataset is available on Mendeley Data (Effrosynidis, 2019).

**Table:** Specifications table

Subject area	Biology
More specific subject area	Marine Ecology
Type of data	Table, figures
How data was acquired	Several datasets from sources such as UNEP-WCMC global biodiversity standardized database, Copernicus Marine Environmental Service, European Marine Observation and Data Network, and Hydrosheds were aggregated using Python.
Data format	Raw, processed, and analyzed.
Experimental factors	The data consists of in-situ observations and the variables were aggregated from several databases.
Experimental features	For each observation, 217 environmental variables are presented
Data source location	Mediterranean Sea (from −5.60° to 36.29° E in longitude and 30.18° to 45.97° N in latitude).
Data accessibility	All data are publicly available on Mendeley Data (https://data.mendeley.com/datasets/8nmh5grxp8/1) [2]
Related research article	Effrosynidis D., Arampatzis A., & Sylaios, G. (2018). Seagrass Detection in the Mediterranean: A Supervised Learning Approach. Ecological Informatics 2018, 48, 158–170, https://doi.org/10.1016/j.ecoinf.2018.09.004[1].

**Table:** 

**Value of the data** • This dataset can be used in machine learning classification tasks to classify an unknown location having seagrass or not, and if it has, in which seagrass family it belongs to. • This dataset can also help in finding which variables are most important when classifying seagrasses and under which conditions each seagrass family favors. • Provided data can be used to resolve conditions related to seagrass presence and absence and the differences between them.

## 1 Data

**Fig. 1:**
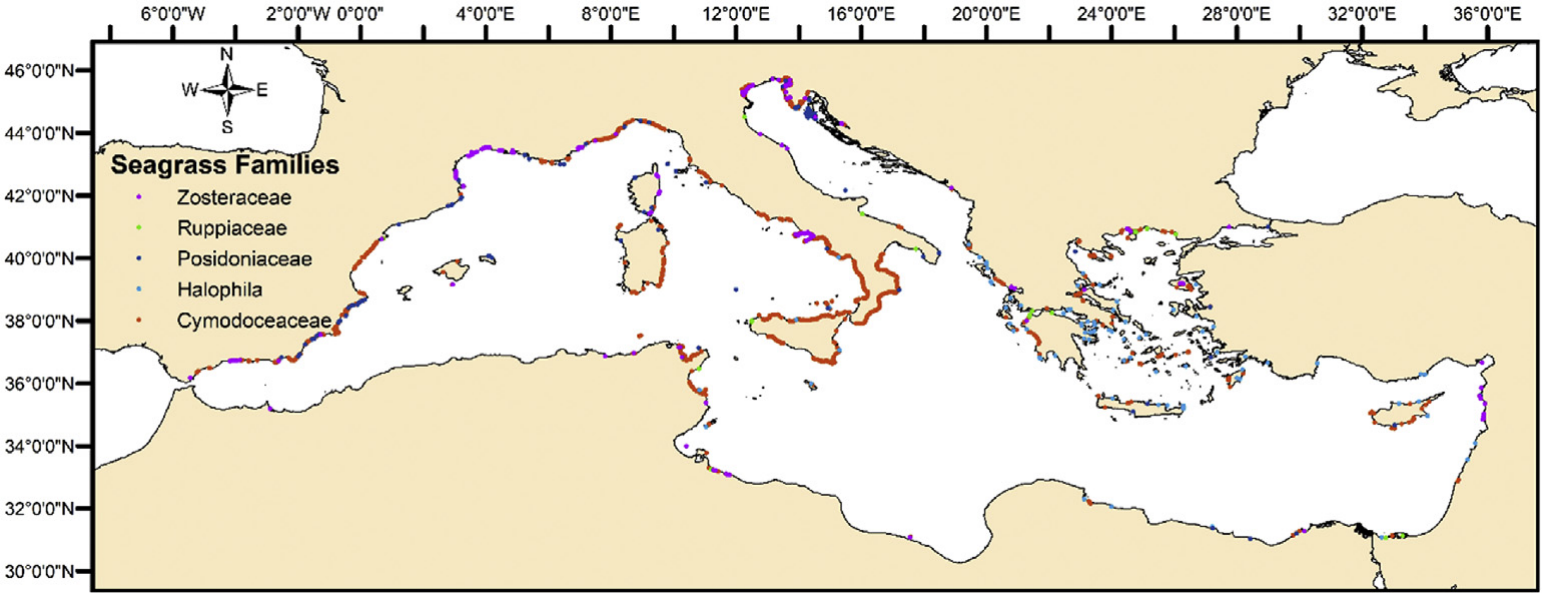
Seagrass Families data points in the Mediterranean Sea.

**Fig. 2:**
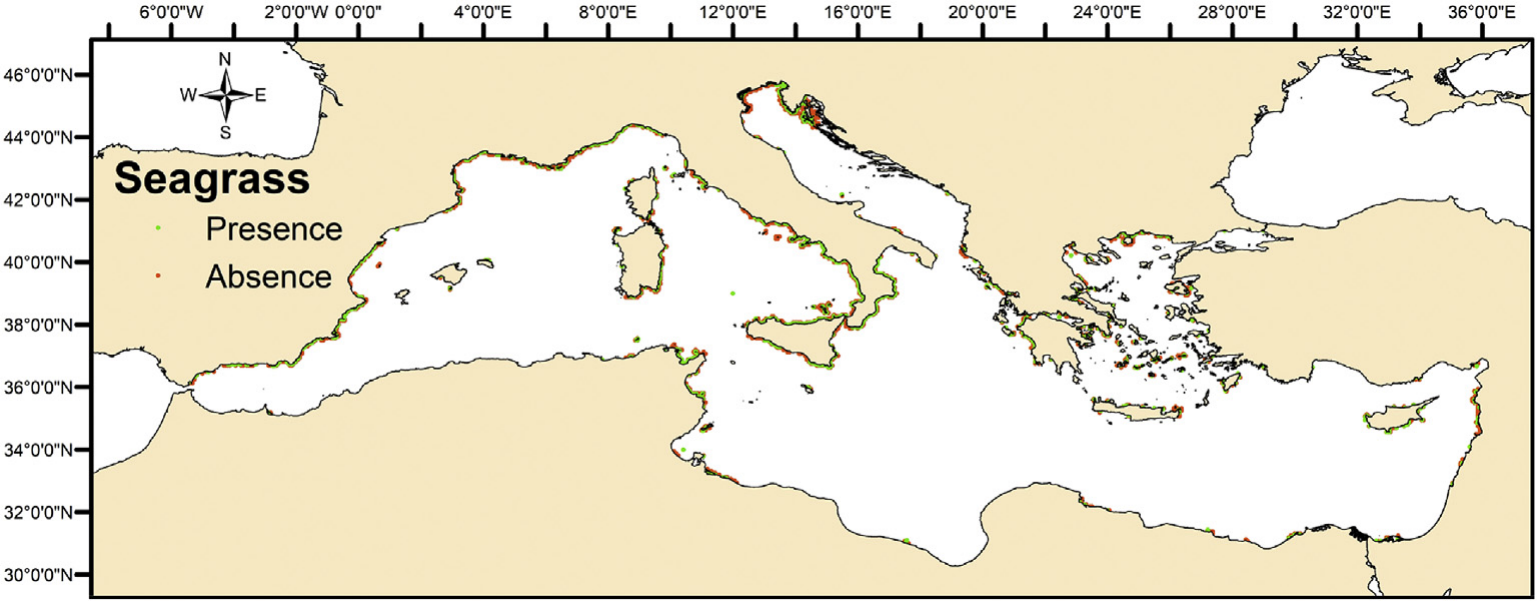
Dataset with seagrass presence and absence points.

The present article delivers data for seagrass presence observations in the Mediterranean Sea (Fig. 1) in combination to a set of parameters (Table 2) describing the local environmental, ecosystemic and human impact forcings. It was produced after geo-locating, sub-setting, aggregating, and compiling “big geo-referenced datasets” maintained from various openly available data sources. This posed a major challenge, especially as these data consisted of blended in-situ observations and assimilated numerical model outcomes, differing in spatial resolution. In addition, a seagrass absence dataset was algorithmically developed (Fig. 2), following certain rules and hypothesis. A Python code was developed following a set of well-defined rules to generate points exhibiting the absence in seagrass meadows.

The dataset has a tabular format, with each row representing an observation point and each column representing the different variables describing this point. There are also two columns that contain the coordinates of each data point, an identification number (ID column), a column that states in which class this entry belongs (absence or the name of the seagrass species), and a similar column that groups species into families (Table 1).

**Table 1:** Seagrass families’ presence in dataset. The last column shows how the species of the initial dataset can be grouped to form the families.

Seagrass Family	Instances	Percentage	Species of this Family
*Cymodoceaceae*	1,337	75.49%	*Cymodocea nodosa, Cymodoceaceae Cymodocea nodosa*
*Zosteraceae*	187	10.56%	*Zostera noltii, Zostera marina, Zosteraceae Zostera noltii, Zosteraceae Zostera marina*
*Posidoniaceae*	125	7.07%	*Posidoniaceae Posidonia oceanica*
*Hydrocharitaceae*	94	5.30%	*Halophila stipulacea, Hydrocharitaceae Halophila stipulacea*
*Ruppiaceae*	28	1.58%	*Ruppia maritima, Ruppia cirrhosa*

**Table 2:** Seagrass families’ Hydrographic Variables selected to be collected from publicly available databases for each seagrass data point.

Name	Type	Layers	Variables
Bathymetry	Static	–	1
Temperature	Temporal	2 (surface, bottom)	38
Salinity	Temporal	2 (surface, bottom)	38
Chlorophyll-α	Temporal	1 (surface)	19
Nitrate	Temporal	2 (surface, bottom)	38
Phosphate	Temporal	2 (surface, bottom)	38
Secchi Disk Depth	Temporal	1 (surface)	19
Wave Height	Temporal	1 (surface)	19
Distance from nearest City	Static	–	2
Distance from nearest River Mouth	Static	–	2
Distance from nearest Port	Static	–	1
Distance to Coast	Static	–	1
Substrate	Static	–	1

Seagrass beds are considered as highly-valued components of coastal ecosystems related to nutrients cycling, carbon sequestration, food-web structure and ecosystem services [3], [4], [5]. Understanding the non-linearities hidden in the relations of seagrass presence with critical physio-chemical factors may aid the assessment of climate change effects.

## 2 Experimental design, materials, and methods

In this section we analyze in detail how we created the dataset using external datasets for the seagrass points and the environmental variables.

### 2.1 Mediterranean sea

The data points for seagrass abundance are located over the Mediterranean Sea, with margins from −5.60° to 36.29° E in longitude and 30.18° to 45.97° N in latitude The initial geo-referenced dataset on seagrass species distribution was provided by the UNEP-WCMC (United Nations Environment Programme - World Conservation Monitoring Centre) [6]. The Mediterranean Sea was selected because of i) the high number of observations compared to other regions, ii) the availability of high spatial resolution variables from external databases, and iii) the fact that there are five major seagrass families present, with one of them (*Hydrocharitaceae*), invading the Mediterranean Sea since the opening of the Suez Channel. However, the same procedure could be followed for any other region on the globe, having sufficient external data to aggregate. In this database a limited number of problematic points was identified, based mostly on the depth zone distribution and the distance to coast. For example, there were some points geo-located at extreme depths, impossible for any seagrass species to live. As these points were very few, and this is a real-world dataset where the measuring instruments are not perfect and imprecise, we did not exclude them and they can be used for outlier analysis. So, all the observations are near the coasts, with few exceptions.

### 2.2 Dataset creation

Several steps were taken to reach the final form of the dataset. The first step was to obtain the seagrass presence reviewed data. The UNEP-WCMC global biodiversity standardized database [6] was used as a source in this scope. This dataset illustrates the global distribution of seagrass species and is given through a geo-referenced shapefile. Data were filtered and only instances located in the Mediterranean Sea were kept (Fig. 1).

The initial dataset had, for each data point, the seagrass species. There were 17 observations with the tag “unspecified” where the name of the seagrass species should be. We excluded these points from our final dataset and created a new column by grouping species into families. The names of the species that were grouped into families can be seen in Table 1.

Next, for each observation point, 217 environmental variables were extracted from several publicly available hydrographic databases, shown in Table 2. Selecting the most appropriate environmental variables is considered an important task in determining the distribution of seagrass taxa. In this work we selected the most appropriate environmental parameters based on an extended literature review and taking the opinion of various seagrass experts. Although, several local/regional studies on the subject exist documenting the impact of environment to the presence and species of seagrass, the relative influence of environmental drivers, ecosystem components and human impacts was never previously reported.

For the extraction of the temporally-changing hydrographic variables, the Copernicus Marine Environmental Service (CMEMS) database [7] was used, as it is the most comprehensive platform for the Mediterranean Sea. It contains re-analyzed hydrographic (water temperature, salinity, currents, waves, etc.) and water quality data (nutrients, dissolved oxygen, chlorophyll-a, etc.) produced in gridded form by numerical models (the hydrodynamic NEMO-OPA and the biogeochemical 3DVAR-OGSTM-BFM models). Model outcomes assimilated the data collected by on-site sensors and satellites. The final products are oceanographic data in netCDF format, annually-updated, covering the whole Mediterranean Sea reporting mean-monthly values per model cell.

These data were imported into a Geographic Information System (QGIS). Monthly mean data were extracted for the year 2015 (considered as a typical year) for both surface and seabed, and the value of the raster pixel that its centre was closer to a seagrass point geo-location was kept. The year 2015 was chosen because it was the most recent year that the external data sources had in common at the time the dataset was created. From this dataset, we get 12 features per layer (surface or bottom), which are the values for each month of the year 2015. We also compute another 4 values which are the mean per season (winter, spring, summer, autumn), and a final 3 which are the min, mean, and max for the year 2015, totaling 12 + 4+3 = 19 variables for one layer. In the cases were seabed values are also considered, the variables are doubled to 38. Based on our experience with machine learning on such data, it seems that features based on seasonal mean, and yearly min, max, provide sufficient granularity and information for learning models. Raw time series, e.g. day-to-day temperature, are less important for seagrass growth than its min/max extremes. Table 3 presents the CMEMS Product used for this dataset and its spatial resolution of the netCDF files.

**Table 3:** CMEMS products and spatial resolution for the temporally-changing hydrographic and water quality variables.

Parameter	CMEMS Product	Resolution
Chlorophyll-a	MEDSEA REANALYSIS_BIO_006_008	0.063° × 0.063°
Nutrients	MEDSEA REANALYSIS_BIO_006_008	0.063° × 0.063°
Salinity	MEDSEA REANALYSIS_PHYS_006_004	0.063° × 0.063°
Secchi Disk depth	OCEANCOLOUR_GLO_OPTICS_L4_REP_OBSERVATIONS_009_081	1 km × 1 km
Significant wave height	MEDSEA_HINDCAST_WAV_006_012	0.042° × 0.042°
Water Temperature	MEDSEA REANALYSIS_PHYS_006_004	0.063° × 0.063°

The static variables were extracted from several sources. The EMODnet (European Marine Observation and Data Network) [8] platform provided the bathymetry and the substrate variables. EMODnet bathymetric data consist of data from bathymetric surveys, hydrographic composite datasets and The General Bathymetric Chart of the Oceans (GEBCO) digital bathymetry data on which digital terrain models (DTMs) have been applied. It is the most reliable and comprehensive dataset presently available for the Mediterranean Sea. Similarly, EMODnet habitat dataset consists of an interoperable product assembling individual point datasets from seabed habitat surveys, maps and models from various sources, aiding to assess the environmental state of ecosystems. The level of data quality varies, with more reliable data those produced by surveys. Same as before, the value that was closer to the seagrass points was extracted for the present dataset.

For the distance-related variables, expressing the human impact on seagrass distribution, external datasets were used. The geo-locations of interest were mapped and the distance of each seagrass observation point to the closest point of human influence (port, coastal city, etc.) was computed using the haversine distance as is the most appropriate because it determines the great-circle distance between two points on a sphere given their longitudes and latitudes. For cities, two datasets were used, one for all major cities [9] and another for all coastal communities.^1^ The same strategy was followed for the distance to river mouths using two additional datasets [9], [10]. A dataset for ports^2^ and a dataset for coasts [11] were finally used to create these fields in our dataset. All distances were reported in degrees as it is the default distance metric in QGIS.

The final step was to clear the dataset from incomplete data and outliers. So, we removed rows that had a significant amount of missing data and replaced the outliers with the average of the column.

### 2.3 Absence dataset

Because there are not publicly available seagrass absence datasets, we proceeded in creating our own following some rules and a hypothesis. We claim that there is a high probability, points near the seagrass presence dataset had already been checked and no seagrass was reported. If there was seagrass, it would had been in the presence dataset already. We do not check for seagrass absence in locations far from locations with seagrass abundance. Therefore, the issue of under-sampling at certain parts of the Mediterranean is not affecting the dataset and the analysis followed. According to our previous research [1], if the artificially created dataset would contain any uncertainty, it would not affect the classification measures significantly, even for a 20% error case.

Based on the above assumption, a Python code was developed and applied on Q-GIS following a set of well-defined rules to generate points exhibiting an absence in seagrass. We used 2 files: the first is the seagrass dataset and the second is the data of any temporal environmental variable obtained from CMEMS. The main purpose of the code is to do comparisons of distances between the longitudes and latitudes of the points of these files in order to discover valid absence points. The distance is calculated using the haversine distance. As CMEMS data are gridded and geo-referenced, environmental data were assigned at the centre point of each CMEMS pixel. As it has already been shown in Table 3, different CMEMS products have different resolutions, so depending on the choice, the absence points can be denser or not. We used the Water Temperature shapefile. At this point we have the longitudes and latitudes of the initial seagrass presence points, and the longitudes and latitudes of the centers of the gridded CMEMS data. For each point of the initial seagrass dataset, we searched for the closest point from the gridded data that has not already found to be in lack of seagrass by previous iterations. A python list was used to keep track of which points were already checked. There is also a restriction that forbids a point to be selected if its distance from the nearest shoreline is longer than 10 km. So, if many seagrass presence points are close to each other in an area, the probability of occurrence of absence points is low. Researchers can follow the same approach to generate their own absence points as it is very easy to implement and requires only two parameters; the choice of the gridded dataset and the distance that restricts points to be considered as absence.

Following the above procedure, 1,284 points of seagrass absence were created and the final dataset consists of a total of 3,055 points as presented in Fig. 2.

### 2.4 Dataset analysis

The final dataset has 3,055 entries with 1,771 records indicating seagrass presence and 1,284 records representing seagrass absence. For each observation, 217 environmental variables are reported. The only categorical variable is the substrate and its values as given by the external dataset are presented in Table 4.

**Table 4:** Substrate Types at final seagrass database points (data from EMODnet database).

Substrate Type	Count
Unknown	663
Sand	550
Fine mud	503
*Posidonia oceanica* meadows	401
Seabed Sandy mud	287
Coarse and mixed sediment	275
Muddy sand	189
Dead mattes of *Posidonia oceanica*	101
*Cymodocea nodosa* meadows	47
Seabed	34
SeabedSeabed	5

The statistics for the other static variables are shown in Table 5. Bathymetry is measured in meters and all other variables in the respective length of a degree of longitude.

**Table 5:** Statistics for static variables.

	Bathymetry (m)	Distance to Major Cities (degrees)	Distance to Complete Cities (degrees)	Distance to Port (degrees)	Distance to Major River (degrees)	Distance to Complete River (degrees)	Distance to Coast (degrees)
Mean	−52.52	0.6843	0.0416	0.2564	2.0753	6.6912e-02	0.0149
Std	123.99	0.4162	0.0457	0.2183	1.6058	8.6419e-02	0.0248
Min	−2416.80	0.0037	0.000015	0.00034	0.0207	8.2400e-11	0
Median	−17.46	0.6604	0.0314	0.19463	1.5440	4.1886e-02	0.0076
Max	0	2.7394	1.0354	1.25	9.8424	7.1247e+00	1.0068

Having many temporal variables, in Table 6 we present only the spatial statistics for the annual average values at sea surface.

**Table 6:** Spatial Statistics for temporally-changing variables (Annual average values at the sea surface).

	Temperature (degC)	Salinity	Chlorophyll-α (mg/m^3^)		Nitrate (mmol/m^3^)	Phosphate (mmol/m^3^)	Secchi Disk Depth (m)	Wave Height (m)
Mean	19.64	38.15		0.21	0.79	0.01	17.05	0.63
Std	1.41	0.99		0.22	2.55	0.01	5.91	0.25
Min	16.31	25.92		0.00	0.00	0.00	1.82	0.12
Median	19.88	38.11		0.13	0.20	0.01	17.78	0.60
Max	23.50	39.60		2.27	38.76	0.46	38.74	1.78

**Fig. 3:**
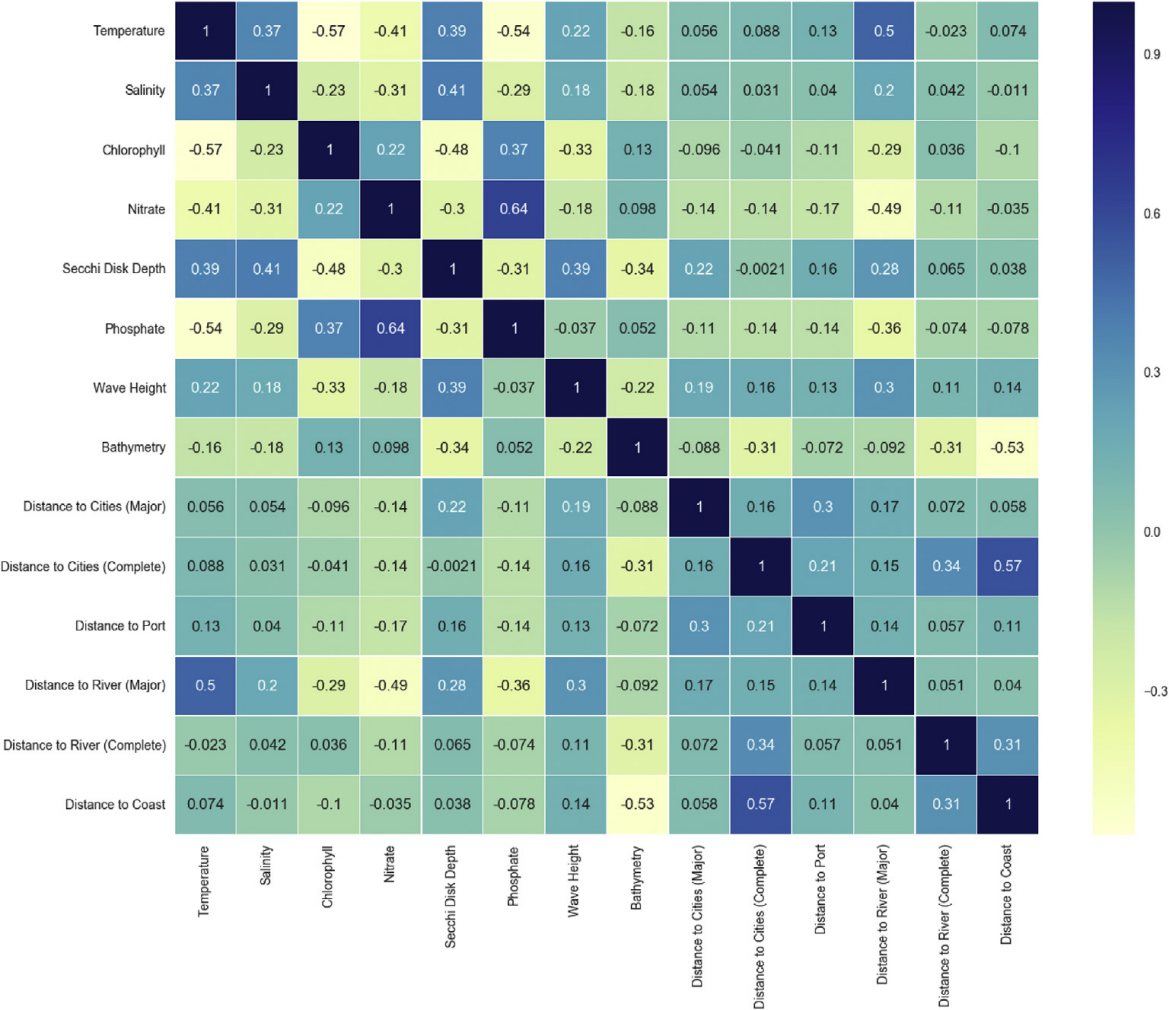
Variable correlation using spearman's rank.

Finally, we display the Spearman's rank between the variables of the dataset as it is a standard first step for machine learning engineers to check for the correlation between variables (Fig. 3). For the temporal variables, the annual average values were used.

## Footnotes

1 https://www.maxmind.com/en/free-world-cities-database.

2 https://msi.nga.mil/NGAPortal/MSI.portal?_nfpb=true&_pageLabel=msi_portal_page_62&pubCode=0015.
